# Supplementary material for: Loss of olfaction reduces caterpillar performance and increases susceptibility to a natural enemy
Source: eLife. 2025 Aug 1;14:RP105585. doi: 10.7554/eLife.105585 (PMC12316457; doi:10.7554/eLife.105585)
Supplement: Supplementary file 1. — (A) Volatile compounds detected in the headspace of the different treatment samples. Cg, Cotesia glomerata female parasitoid wasps (n = 12); Pb, Pieris brassicae caterpillars (n = 10); Pb–Cg, P. brassicae caterpillars in the presence of C. glomerata female parasitoid wasps (n = 14); Pb-Fr, P. brassicae caterpillar frass (n = 10); and Pb-S, P. brassicae spit (n = 12), are listed. Relative amounts of volatiles are presented as average peak height (SE)/104. The volatiles are listed according to their elution order in a chromatographic window. Volatiles with variable importance in the projection (VIP) scores equal to or higher than 1.0 (presented in bold) are considered important in separating the different treatment groups of the given analysis. Significant differences among the five treatments detected by the Kruskal–Wallis test with Dunn’s post hoc test for multiple comparisons are indicated by letters in the table. Different letters indicate significant difference (p < 0.05). NF, not found. (B) Volatiles used in separating C. glomerata female wasps (Cg, n = 12), P. brassicae caterpillars (Pb, n = 10), and the interaction of P. brassicae caterpillars with C. glomerata female wasps (Pb–Cg, n = 14) sample treatments (Figure 3—figure supplement 1). Volatiles are listed according to ranking order of their VIP score values, where those with VIP scores of equal to or higher than 1.0 are considered important in separating the treatment groups of the given analysis. (C) Volatiles used in separating P. brassicae caterpillars (Pb, n = 10) and the interaction of P. brassicae caterpillars with C. glomerata female wasps (Pb–Cg, n = 14) sample treatments (Figure 3—figure supplement 2). Volatiles are listed according to ranking order of their VIP score values, where those with VIP scores of equal to or higher than 1.0 are considered important in separating the treatment groups of the given analysis. (D) Volatiles used in separating the interaction of P. brassicae caterpillars with [file elife-105585-supp1.pdf]

Loss of olfaction reduces caterpillar performance and increases susceptibility to a natural enemy

https://doi.org/10.7554/eLife.105585.2

Qi Wang [1], Yufei Jia [1], Hans M. Smid [1], Berhane T. Weldegergis [1], Liana O. Greenberg [2], Maarten Jongsma [3], Marcel Dicke [1], Alexander Haverkamp [1,4]*

[1] Laboratory of Entomology, Wageningen University & Research, Wageningen, the Netherlands

[2] Biosystematics Group, Wageningen University & Research, Wageningen, the Netherlands

[3] Business Unit Bioscience, Wageningen University & Research, Wageningen, the Netherlands

[4] Lead contact

* Correspondence: alexander.haverkamp@wur.nl (A.H.)

Table of Contents

Table A. Volatile compounds detected in the headspace of the different treatment samples.

Table B. Volatiles used in separating *Cotesia glomerata* female wasps (Cg, n = 12), *Pieris brassicae* caterpillars (Pb, n = 10) and the interaction of *P. brassicae* caterpillars with *C. glomerata* female wasps (Pb-Cg, n = 14) sample treatments (Figure 3-figure supplement 1).

Table C. Volatiles used in separating *Pieris brassicae* caterpillars (Pb, n = 10) and the interaction of *P. brassicae* caterpillars with *C. glomerata* female wasps (Pb-Cg, n = 14) sample treatments (Figure 3-figure supplement 2).

Table D. Volatiles used in separating the interaction of *Pieris brassicae* caterpillars with *Cotesia glomerata* female wasps (Pb-Cg, n = 14) and *P. brassicae* caterpillar spit (Pb-S, n = 12), sample treatments (Figure 3-figure supplement 3).

Table E. Volatiles used in separating the *Pieris brassicae* caterpillar spit (Pb-S, n = 12), and *P. brassicae* caterpillar frass (Pb-Fr, n = 10) sample treatments (Figure 3-figure supplement 4).

Table F. Volatiles used in separating the *Pieris brassicae* caterpillars (Pb, n = 10), the interaction of *P. brassicae* caterpillars with *Cotesia glomerata* female wasps (Pb-Cg, n = 14), and *P. brassicae* caterpillar frass (Pb-Fr, n = 10) sample treatments (Figure 3-figure supplement 5).

Table G. Chemical compounds that were used for the electroantennographical test.

Table H. Chemical compounds that were used for the behavioral test in multi-channel arena.

Table A. Volatile compounds detected in the headspace of the different treatment samples. Cg, *Cotesia glomerata* female parasitoid wasps (n = 12); Pb, *Pieris brassicae* caterpillars (n = 10); Pb-Cg, *P. brassicae* caterpillars in the presence of *C. glomerata* female parasitoid wasps (n = 14); Pb-Fr, *P. brassicae* caterpillar frass (n = 10); and Pb-S, *P. brassicae* spit (n = 12), are listed. Relative amounts of volatiles are presented as average peak height (SE)/10^4. The volatiles are listed according to their elution order in a chromatographic window. Volatiles with Variable Importance in the Projection (VIP) scores equal to or higher than 1.0 (presented in bold), are considered important in separating the different treatment groups of the given analysis. Significant differences among the five treatments detected by Kruskal-Wallis test with Dunn's post-hoc test for multiple comparisons are indicated by letters in the table. Different letters indicate significant difference (P < 0.05). NF, not found.

Number	Compound and class	Cg	Pb	Pb-Cg	Pb-S	Pb-Fr	VIP-SCORE
1	2,3-Butanedione	9.9 (1.2) a	115.9 (17.7) b	290.9 (106.5) b	167.1 (33.1) b	1540.7 (712.9) b	**1.04**
2	2-Butenenitrile	NF a	NF a	1.2 (0.6) a	11.3 (3) b	NF a	**1.17**
3	3-Methylbutanal	0.1 (0.1) a	2 (0.4) ab	3.4 (0.5) bc	243.4 (21.1) d	92.1 (31.5) cd	0.91
4	2-Methylbutanal	0.1 (0.1) a	1.6 (0.3) ab	3.2 (0.5) bc	277.9 (23.7) d	108.1 (36.6) cd	0.91
5	1-Methoxy-2-propanol	3.1 (1.3) a	74.8 (15.3) b	256.5 (93.6) b	149.2 (9.6) b	538.2 (259.6) b	**1.04**
6	1-Penten-3-ol	NF a	55 (11.9) ab	158.7 (51.8) b	466.6 (64.3) c	1028.1 (414) bc	0.98
7	Pentanal	7.2 (0.8) a	8.4 (1.1) a	9.6 (2.6) a	358.6 (29.8) b	22.9 (12) a	**1.11**
8	Methyl thiocyanate	NF a	NF a	NF a	69.5 (7.9) b	86.6 (31.9) b	0.98
9	3-Methyl-1-butanol	NF a	NF a	1.6 (1.2) a	82.9 (39.7) b	90.4 (44.3) b	0.78
10	3-Penten-2-one	1.2 (0.6) a	2.6 (1.1) a	4.5 (1.6) a	104.3 (26.6) b	122 (50.3) b	0.82
11	Unknown	NF a	0.2 (0.2) a	0.3 (0.2) a	62.8 (5.7) b	324.4 (162.9) ab	0.85
12	Dimethyl disulfide	1.5 (0.6) a	182.5 (23.9) ab	780.2 (131.3) bc	2738 (296.5) c	3182.7 (589) c	**1.10**
13	(Z)-2-Pentenal	NF a	1.4 (0.6) a	3.1 (0.8) ab	102.2 (7.5) c	36.4 (14.8) bc	0.86
14	3-Methyl-3-butenenitrile	NF a	NF a	1 (0.4) a	12.4 (2.7) b	0.2 (0.1) a	**1.30**
15	Methylthioacetaldehyde	NF a	NF a	NF a	0.1 (0.1) a	90.9 (32.1) b	**1.40**
16	1-Pentanol	1.7 (0.9) a	1.5 (0.8) a	3.7 (1.5) ab	220.8 (56.8) c	58.1 (23.1) bc	0.81
17	(Z)-2-Penten-1-ol	NF a	NF a	0.6 (0.4) a	63.4 (7.8) b	40.1 (18) b	0.87
18	2,3-Butanediol	NF a	1.1 (1.1) a	19.5 (10.6) ab	23.4 (5.4) b	187.8 (94.9) ab	0.93
19	Ethyl methanesulfinate	NF a	NF a	NF a	31.4 (8.8) b	11.3 (3.7) b	0.95
20	3-Methylbutanoic acid	NF a	NF a	0.3 (0.1) a	18.7 (1.8) b	3.1 (1.1) ab	0.89
21	2,3-Heptanedione	NF a	NF a	NF a	4.6 (0.5) b	NF a	**1.14**
22	(Z)-3-Hexen-1-ol	2.3 (1.5) a	42.2 (5.3) ab	98.7 (23.4) b	3384.9 (454.5) c	1332.7 (569.4) bc	0.97
23	(Z)-2-Hexen-1-ol	NF a	NF a	0.6 (0.5) a	99.8 (22.7) b	15.5 (7.6) a	0.94
24	1-Hexanol	0.3 (0.3) a	NF a	2.3 (1.3) a	70.8 (21.6) b	43.5 (17.5) b	0.86
25	Cyclohexanol	NF a	9 (2.8) b	25.4 (7.1) b	4 (1.2) ab	397.9 (197.6) b	**1.06**
26	3-Ethyl-1,5-octadiene, Isomer I	NF a	13.3 (1.8) b	10.9 (1.8) b	0.2 (0.2) a	NF a	**1.15**
27	3-Ethyl-1,5-octadiene, Isomer II	NF a	53.7 (7.3) b	48.5 (6.5) b	NF a	NF a	**1.18**
28	3-(Methylthio)propanal	NF a	NF a	NF a	24.5 (3.3) b	6.2 (3.2) a	0.99
29	Unknown	NF a	NF a	NF a	42 (7.4) b	3.6 (2.4) a	0.97
30	3,7-Decadiene, Isomer I	NF a	9.9 (0.8) bc	12.2 (2.6) bc	19.8 (3.9) c	2.8 (1.1) ab	**1.20**
31	Dimethyl trisulfide	NF a	19 (2.9) ab	249.4 (48.4) bc	6948 (557.9) d	2099.9 (354.5) cd	**1.06**
32	3,7-Decadiene, Isomer II	NF a	6.3 (1.3) bc	9.9 (2.4) c	23 (5.2) c	1.2 (0.9) ab	**1.04**
33	(E,E)-2,4-Heptadienal	NF a	NF a	NF b	5.9 (1.1) b	5.8 (2.2) b	0.86
34	Phenylacetaldehyde	5.1 (0.4) a	5 (0.5) a	6.8 (1) a	38.5 (3.5) b	11.6 (3) a	0.94
35	3-Methyl-2-butenyl 2-methylbutanoate	0.8 (0.5) a	NF a	NF a	140.3 (8.5) b	56.5 (23.9) b	0.94
36	Methyl (methylthio)methyl disulfide	NF a	0.2 (0.1) a	6.7 (2) b	76.3 (7.9) c	72.5 (28.6) bc	**1.19**
37	Benzyl cyanide	1.3 (0.3) a	2.8 (0.6) ab	10.7 (2.2) bc	549.6 (66.4) d	31 (11) cd	0.95
38	4-Ketoisophorone	NF a	NF a	NF a	3.9 (0.3) b	7.7 (3.4) b	0.94
39	beta-Cyclocitral	NF a	0.4 (0.1) a	0.6 (0.2) a	28.2 (1.4) b	77.8 (28.7) b	0.93
40	Dimethyl tetrasulfide	NF a	NF a	0.6 (0.2) a	2119.4 (226.8) b	145.5 (36) b	0.90
41	Chavibetol	NF a	0.3 (0.2) a	1.9 (1) a	30.1 (3.5) b	77.6 (37.2) b	0.87
42	(E)-beta-Ionone	NF a	0.5 (0.5) a	3 (1.1) a	104.8 (5) b	145.9 (53.5) b	0.91
43	beta-Ionone epoxide	NF a	0.5 (0.3) a	1.3 (0.4) a	51 (3.4) b	44.6 (17.8) b	0.89
44	Dihydroactinidiolide	NF a	0.1 (0.1) a	0.2 (0.1) a	4.7 (0.5) b	3.4 (1.4) b	0.79
45	Tricyclopentadeca-3,7-dien[8.4.0.1(11,14)]	0.7 (0.2) a	10.9 (0.7) b	8.8 (0.9) b	3.4 (0.2) a	1.6 (0.6) a	**1.20**

Table B. Volatiles used in separating *Cotesia glomerata* female wasps (Cg, n = 12), *Pieris brassicae* caterpillars (Pb, n = 10) and the interaction of *P. brassicae* caterpillars with *C. glomerata* female wasps (Pb-Cg, n = 14) sample treatments (Figure 3-figure supplement 1). Volatiles are listed according to ranking order of their Variable Importance in the Projection (VIP) scores values, where those with VIP scores of equal to or higher than 1.0, are considered important in separating the treatment groups of the given analysis.

Primary ID	Chemical name	VIP-Score
36	Methyl (methylthio)methyl disulfide	1.56332
31	Dimethyl trisulfide	1.39172
14	3-Methyl-3-butenenitrile	1.37969
27	3-Ethyl-1,5-octadiene, Isomer II	1.37419
30	3,7-Decadiene, Isomer I	1.35815
26	3-Ethyl-1,5-octadiene, Isomer I	1.34633
12	Dimethyl disulfide	1.31333
6	1-Penten-3-ol	1.29147
25	Cyclohexanol	1.26832
32	3,7-Decadiene, Isomer II	1.26563
45	Tricyclopentadeca-3,7-dien[8.4.0.1(11,14)]	1.26062
3	3-Methylbutanal	1.21712
4	2-Methylbutanal	1.20836
22	(Z)-3-Hexen-1-ol	1.20629
5	1-Methoxy-2-propanol	1.19415
1	2,3-Butanedione	1.1492
40	Dimethyl tetrasulfide	1.09912
37	Benzyl cyanide	1.09685
24	1-Hexanol	1.06427
2	2-Butenenitrile	1.06105
13	(Z)-2-Pentenal	0.978154
11	Unknown	0.974729
17	(Z)-2-Penten-1-ol	0.965661
39	beta-Cyclocitral	0.942238
43	beta-Ionone epoxide	0.933374
9	3-Methyl-1-butanol	0.932564
41	Chavibetol	0.928908
34	Phenylacetaldehyde	0.832774
18	2,3-Butanediol	0.826549
42	(E)-beta-Ionone	0.809716
33	(E,E)-2,4-Heptadienal	0.705911
20	3-Methylbutanoic acid	0.663228
28	3-(Methylthio)propanal	0.652614
23	(Z)-2-Hexen-1-ol	0.635438
21	2,3-Heptanedione	0.633611
38	4-Ketoisophorone	0.62339
7	Pentanal	0.575643
10	3-Penten-2-one	0.557406
35	3-Methyl-2-butenyl 2-methylbutanoate	0.554326
8	Methyl thiocyanate	0.526719
19	Ethyl methanesulfinate	0.514083
16	1-Pentanol	0.505486
44	Dihydroactinidiolide	0.502176
15	Methylthioacetaldehyde	0.476651
29	Unknown	0.132699

Table C. Volatiles used in separating *Pieris brassicae* caterpillars (Pb, n = 10) and the interaction of *P. brassicae* caterpillars with *C. glomerata* female wasps (Pb-Cg, n = 14) sample treatments (Figure 3-figure supplement 2). Volatiles are listed according to ranking order of their Variable Importance in the Projection (VIP) scores values, where those with VIP scores of equal to or higher than 1.0, are considered important in separating the treatment groups of the given analysis.

Primary ID	Chemical name	VIP-Score
36	Methyl (methylthio)methyl disulfide	2.07587
12	Dimethyl disulfide	1.92275
31	Dimethyl trisulfide	1.69443
14	3-Methyl-3-butenenitrile	1.5985
37	Benzyl cyanide	1.51645
24	1-Hexanol	1.27124
35	3-Methyl-2-butenyl 2-methylbutanoate	1.24411
40	Dimethyl tetrasulfide	1.23794
2	2-Butenenitrile	1.18014
45	Tricyclopentadeca-3,7-dien[8.4.0.1(11,14)]	1.16026
11	Unknown	1.11114
17	(Z)-2-Penten-1-ol	1.06676
9	3-Methyl-1-butanol	1.03313
41	Chavibetol	0.999371
43	beta-Ionone epoxide	0.98264
18	2,3-Butanediol	0.957447
34	Phenylacetaldehyde	0.942159
13	(Z)-2-Pentenal	0.916875
28	3-(Methylthio)propanal	0.909736
39	beta-Cyclocitral	0.90317
6	1-Penten-3-ol	0.890276
4	2-Methylbutanal	0.877197
22	(Z)-3-Hexen-1-ol	0.857471
42	(E)-beta-Ionone	0.851761
26	3-Ethyl-1,5-octadiene, Isomer I	0.842136
1	2,3-Butanedione	0.839521
3	3-Methylbutanal	0.831809
5	1-Methoxy-2-propanol	0.81872
25	Cyclohexanol	0.785347
30	3,7-Decadiene, Isomer I	0.783212
38	4-Ketoisophorone	0.746923
33	(E,E)-2,4-Heptadienal	0.728538
21	2,3-Heptanedione	0.71051
23	(Z)-2-Hexen-1-ol	0.710482
20	3-Methylbutanoic acid	0.683183
27	3-Ethyl-1,5-octadiene, Isomer II	0.642632
7	Pentanal	0.631077
16	1-Pentanol	0.605405
32	3,7-Decadiene, Isomer II	0.590844
19	Ethyl methanesulfinate	0.557555
8	Methyl thiocyanate	0.55321
10	3-Penten-2-one	0.465652
29	Unknown	0.322982
44	Dihydroactinidiolide	0.273144
15	Methylthioacetaldehyde	0.0785777

Table D. Volatiles used in separating the interaction of *Pieris brassicae* caterpillars with *Cotesia glomerata* female wasps (Pb-Cg, n = 14) and *P. brassicae* caterpillar spit (Pb-S, n = 12), sample treatments (Figure 3-figure supplement 3). Volatiles are listed according to ranking order of their Variable Importance in the Projection (VIP) scores values, where those with VIP scores of equal to or higher than 1.0, are considered important in separating the treatment groups of the given analysis.

Primary ID	Chemical name	VIP-Score
35	3-Methyl-2-butenyl 2-methylbutanoate	1.18987
8	Methyl thiocyanate	1.18927
38	4-Ketoisophorone	1.18925
21	2,3-Heptanedione	1.18687
28	3-(Methylthio)propanal	1.18366
40	Dimethyl tetrasulfide	1.16523
37	Benzyl cyanide	1.164
11	Unknown	1.15829
31	Dimethyl trisulfide	1.15172
23	(Z)-2-Hexen-1-ol	1.13638
17	(Z)-2-Penten-1-ol	1.13498
20	3-Methylbutanoic acid	1.12993
34	Phenylacetaldehyde	1.12219
4	2-Methylbutanal	1.11885
39	beta-Cyclocitral	1.11868
19	Ethyl methanesulfinate	1.11794
3	3-Methylbutanal	1.1088
43	beta-Ionone epoxide	1.07481
16	1-Pentanol	1.05818
42	(E)-beta-Ionone	1.05223
29	Unknown	1.04776
33	(E,E)-2,4-Heptadienal	1.04304
27	3-Ethyl-1,5-octadiene, Isomer II	1.03943
41	Chavibetol	1.03686
13	(Z)-2-Pentenal	1.03618
36	Methyl (methylthio)methyl disulfide	1.01861
12	Dimethyl disulfide	1.00794
22	(Z)-3-Hexen-1-ol	1.00726
44	Dihydroactinidiolide	1.0004
45	Tricyclopentadeca-3,7-dien[8.4.0.1(11,14)]	0.996387
7	Pentanal	0.994438
14	3-Methyl-3-butenenitrile	0.974921
10	3-Penten-2-one	0.971786
26	3-Ethyl-1,5-octadiene, Isomer I	0.935729
24	1-Hexanol	0.901818
6	1-Penten-3-ol	0.856495
2	2-Butenenitrile	0.855662
9	3-Methyl-1-butanol	0.795921
25	Cyclohexanol	0.680023
1	2,3-Butanedione	0.612927
5	1-Methoxy-2-propanol	0.589883
18	2,3-Butanediol	0.513856
30	3,7-Decadiene, Isomer I	0.490609
32	3,7-Decadiene, Isomer II	0.399193
15	Methylthioacetaldehyde	0.293689

Table E. Volatiles used in separating the *Pieris brassicae* caterpillar spit (Pb-S, n = 12), and *P. brassicae* caterpillar frass (Pb-Fr, n = 10) sample treatments (Figure 3-figure supplement 4). Volatiles are listed according to ranking order of their Variable Importance in the Projection (VIP) scores values, where those with VIP scores of equal to or higher than 1.0, are considered important in separating the treatment groups of the given analysis.

Primary ID	Chemical name	VIP-Score
21	2,3-Heptanedione	1.47069
14	3-Methyl-3-butenenitrile	1.41146
37	Benzyl cyanide	1.37845
15	Methylthioacetaldehyde	1.3601
34	Phenylacetaldehyde	1.26864
2	2-Butenenitrile	1.26709
40	Dimethyl tetrasulfide	1.22517
28	3-(Methylthio)propanal	1.2005
13	(Z)-2-Pentenal	1.19752
23	(Z)-2-Hexen-1-ol	1.19502
20	3-Methylbutanoic acid	1.17384
3	3-Methylbutanal	1.17326
4	2-Methylbutanal	1.15938
7	Pentanal	1.15138
31	Dimethyl trisulfide	1.15035
22	(Z)-3-Hexen-1-ol	1.13272
45	Tricyclopentadeca-3,7-dien[8.4.0.1(11,14)]	1.11074
30	3,7-Decadiene, Isomer I	1.09918
29	Unknown	1.08182
32	3,7-Decadiene, Isomer II	1.04475
17	(Z)-2-Penten-1-ol	1.03121
16	1-Pentanol	1.0235
35	3-Methyl-2-butenyl 2-methylbutanoate	1.01617
43	beta-Ionone epoxide	0.971688
11	Unknown	0.952159
36	Methyl (methylthio)methyl disulfide	0.924777
38	4-Ketoisophorone	0.854173
25	Cyclohexanol	0.853377
1	2,3-Butanedione	0.847814
39	beta-Cyclocitral	0.843729
8	Methyl thiocyanate	0.841366
42	(E)-beta-Ionone	0.82494
41	Chavibetol	0.822314
5	1-Methoxy-2-propanol	0.803075
10	3-Penten-2-one	0.765907
19	Ethyl methanesulfinate	0.764512
33	(E,E)-2,4-Heptadienal	0.702099
6	1-Penten-3-ol	0.647433
12	Dimethyl disulfide	0.610209
9	3-Methyl-1-butanol	0.607502
18	2,3-Butanediol	0.570747
24	1-Hexanol	0.567647
44	Dihydroactinidiolide	0.52246
26	3-Ethyl-1,5-octadiene, Isomer I	0.333459
27	3-Ethyl-1,5-octadiene, Isomer II	0.191552

Table F. Volatiles used in separating the *Pieris brassicae* caterpillars (Pb, n = 10), the interaction of *P. brassicae* caterpillars with *Cotesia glomerata* female wasps (Pb-Cg, n = 14), and *P. brassicae* caterpillar frass (Pb-Fr, n = 10) sample treatments (Figure 3-figure supplement 5). Volatiles are listed according to ranking order of their Variable Importance in the Projection (VIP) scores values, where those with VIP scores of equal to or higher than 1.0, are considered important in separating the treatment groups of the given analysis.

Primary ID	Chemical name	VIP-Score
14	3-Methyl-3-butenenitrile	1.53713
36	Methyl (methylthio)methyl disulfide	1.49888
37	Benzyl cyanide	1.44649
31	Dimethyl trisulfide	1.37488
12	Dimethyl disulfide	1.27985
8	Methyl thiocyanate	1.16994
15	Methylthioacetaldehyde	1.16173
35	3-Methyl-2-butenyl 2-methylbutanoate	1.12286
19	Ethyl methanesulfinate	1.12207
38	4-Ketoisophorone	1.09766
40	Dimethyl tetrasulfide	1.08304
2	2-Butenenitrile	1.0747
21	2,3-Heptanedione	1.06918
39	beta-Cyclocitral	1.032
27	3-Ethyl-1,5-octadiene, Isomer II	1.03194
33	(E,E)-2,4-Heptadienal	0.999281
42	(E)-beta-Ionone	0.991173
26	3-Ethyl-1,5-octadiene, Isomer I	0.989933
24	1-Hexanol	0.984174
45	Tricyclopentadeca-3,7-dien[8.4.0.1(11,14)]	0.970013
4	2-Methylbutanal	0.965192
17	(Z)-2-Penten-1-ol	0.958219
43	beta-Ionone epoxide	0.958126
3	3-Methylbutanal	0.940685
41	Chavibetol	0.93559
9	3-Methyl-1-butanol	0.927445
10	3-Penten-2-one	0.892843
20	3-Methylbutanoic acid	0.889393
22	(Z)-3-Hexen-1-ol	0.888146
18	2,3-Butanediol	0.880791
11	Unknown	0.866956
28	3-(Methylthio)propanal	0.852439
13	(Z)-2-Pentenal	0.84884
32	3,7-Decadiene, Isomer II	0.840748
44	Dihydroactinidiolide	0.821653
23	(Z)-2-Hexen-1-ol	0.818063
1	2,3-Butanedione	0.807042
5	1-Methoxy-2-propanol	0.78006
34	Phenylacetaldehyde	0.774451
7	Pentanal	0.767969
16	1-Pentanol	0.766034
30	3,7-Decadiene, Isomer I	0.764335
25	Cyclohexanol	0.72219
29	Unknown	0.695
6	1-Penten-3-ol	0.662903

Table G. Chemical compounds that were used for the electroantennographical test.

Chemical compound	CAS number	Purity	Manufacturer
Acetic acid	64-19-7	⩾ 99.0%	Sigma-Aldrich
Benzoic acid	65-85-0	⩾99.5%	Sigma-Aldrich
Isovaleric acid	503-74-2	99.00%	Sigma-Aldrich
n-Caproic acid	142-62-1	⩾ 99.0%	Sigma-Aldrich
(*E*)-Anethole	4180-23-8	99.00%	Sigma-Aldrich
1,8-Cineole	470-82-6	99.00%	Sigma-Aldrich
1-Hexanol	111-27-3	98.00%	Fluka
1-Methoxy-2-propanol	107-98-2	⩾ 99.5%	Sigma-Aldrich
1-Octen-3-ol	3391-86-4	98.00%	Sigma-Aldrich
1-Pentanol	71-41-0	⩾ 99.0%	Sigma-Aldrich
1-Penten-3-ol	616-25-1	99.00%	Sigma-Aldrich
3-Methyl-1-butanol	123-51-3	⩾ 99.0%	Sigma-Aldrich
3-Octanol	589-98-0	99.00%	Sigma-Aldrich
3-Pentanol	584-02-1	98.00%	Sigma-Aldrich
(*Z*)-2-Penten-1-ol	1576-95-0	95.00%	Sigma-Aldrich
(*Z*)-3-Hexen-1-ol	928-96-1	98.00%	Sigma-Aldrich
Geraniol	106-24-1	98.00%	Sigma-Aldrich
Linalool	78-70-6	97.00%	Sigma-Aldrich
Phenylethyl alcohol	8-12-1960	⩾99.0 %	Fluka
(*E*)-2-Hexen-1-ol	928-95-0	⩾ 95.0%	Sigma-Aldrich
1-Nonanal	124-19-6	95.00%	Sigma-Aldrich
2-Methylbutanal	96-17-3	95.00%	Sigma-Aldrich
3-Methylbutanal	590-86-3	⩾ 98.5%	Fluka
Benzaldehyde	100-52-7	⩾99.0%	Fluka
(*Z*)-2-Pentenal	1576-87-0	95.00%	Sigma-Aldrich
Heptaldehyde	111-71-7	95.00%	Sigma-Aldrich
Hexanal	66-25-1	98.00%	Sigma-Aldrich
Phenylacetaldehyde	122-78-1	⩾95.0%	Fluka
(*E*)-2-Hexenal	6728-26-3	98.00%	Sigma-Aldrich
Nonane	111-84-2	99.00%	Sigma-Aldrich
α-Pinene	80-56-8	98.00%	Sigma-Aldrich
β-Caryophyllene	87-44-5	⩾ 98.0%	Sigma-Aldrich
β-Pinene	127-91-3	⩾ 95.0%	Sigma-Aldrich
Limonene	5989-27-5	97.00%	Sigma-Aldrich
Myrcene	123-35-3	⩾ 99.0	Sigma-Aldrich
(*Z*)-3-Hexenyl acetate	3681-71-8	98.00%	Sigma-Aldrich
Hexyl acetate	142-92-7	99.00%	Sigma-Aldrich
Methyl salicylate	119-36-8	⩾ 98.0%	Sigma-Aldrich
Pentyl acetate	628-63-7	99.00%	Sigma-Aldrich
Benzothiazole	95-16-9	⩾ 96.0%	Sigma-Aldrich
Indole	120-72-9	⩾ 99.0%	Sigma-Aldrich
Allyl ITC	7-6-1957	⩾ 95.0%	Sigma-Aldrich
Benzyl ITC	622-78-6	98.00%	Sigma-Aldrich
Butyl ITC	592-82-5	99.00%	Sigma-Aldrich
Methyl ITC	556-61-6	97.00%	Fluka
Phenyl ITC	103-72-0	98.00%	Sigma-Aldrich
2,3-Butanedione	431-03-8	97.00%	Fluka
3-Hydroxy-2-butanone	513-86-0	⩾ 98.0%	Sigma-Aldrich
3-Octanone	106-68-3	⩾ 98.0%	Sigma-Aldrich
3-Pentanone	96-22-0	⩾ 99.0%	Sigma-Aldrich
3-Butenenitrile	109-75-1	98.00%	Sigma-Aldrich
Benzyl cyanide	140-29-4	98.00%	Sigma-Aldrich
Dimethyl disulfide	624-92-0	⩾ 99.0%	Sigma-Aldrich
Paraffin oil	8012-95-1	-	Sigma-Aldrich

Table H. Chemical compounds that were used for the behavioral test in multi-channel arena.

Chemical compound	CAS number	Purity	Manufacturer
1-Hexanol	111-27-3	98.00%	Fluka
1-Methoxy-2-propanol	107-98-2	≥ 99.5%	Sigma-Aldrich
1-Penten-3-ol	616-25-1	99.00%	Sigma-Aldrich
2,3-Butanedione	431-03-8	97.00%	Fluka
3-Methylbutanal	123-51-3	≥ 98.5%	Fluka
Benzyl cyanide	140-29-4	98.00%	Sigma-Aldrich
Dimethyl disulfide	624-92-0	≥ 99.0%	Sigma-Aldrich
Pentanal	110-62-3	≥ 99.0%	Sigma-Aldrich
(*E*)-2-Hexen-1-ol	928-95-0	≥ 95.0%	Sigma-Aldrich
(*Z*)-2-Penten-1-ol	1576-95-0	95.00%	Sigma-Aldrich
(*Z*)-3-Hexen-1-ol	928-96-1	98.00%	Sigma-Aldrich
Linalool	78-70-6	97.00%	Sigma-Aldrich
(*Z*)-3-Hexenyl acetate	3681-71-8	98.00%	Sigma-Aldrich
Hexanal	66-25-1	98.00%	Sigma-Aldrich
(*E*)-2-Hexenal	6728-26-3	98.00%	Sigma-Aldrich
