# Supplementary material for: Loss of olfaction reduces caterpillar performance and increases susceptibility to a natural enemy
Source: eLife. 2025 Aug 1;14:RP105585. doi: 10.7554/eLife.105585 (PMC12316457; doi:10.7554/eLife.105585)
Supplement: Peer review history

# Editor report: eLife Assessment

**DOI:** 10.7554/eLife.105585.3.sa0

**Authors:** Youngsung Joo

**Affiliations:** https://ror.org/04h9pn542 Seoul National University Republic of Korea

**Keywords:** Convincing, Important

This **important** manuscript investigates the role of olfactory cues in *Pieris brassicae* larvae, focusing on their interactions with the host plant *Brassica oleracea* and the parasitoid wasp *Cotesia glomerata*. The authors' demonstration that impaired olfactory perception reduces caterpillar performance and increases susceptibility to parasitism is **convincing**. These findings highlight the ecological significance of olfaction in mediating feeding behavior and predator avoidance in herbivorous insects.

# Referee report: Reviewer #1 (Public review)

**DOI:** 10.7554/eLife.105585.3.sa1

Summary:

The manuscript focuses on the olfactory system of Pieris brassicae larvae and the importance of olfactory information in their interactions with the host plant Brassica oleracea and the major parasitic wasp Cotesia glomerata. The authors used CRISPR/Cas9 to knockout odorant receptor co-receptors (Orco), and conducted a comparative study on the behavior and olfactory system of the mutant and wild-type larvae. The study found that Orco-expressing olfactory sensory neurons in antennae and maxillary palps of Orco knockout (KO) larvae disappeared, and the number of glomeruli in the brain decreased, which impairs the olfactory detection and primary processing in the brain. Orco KO caterpillars show weight loss and loss of preference for optimal food plants; KO larvae also lost weight when attacked by parasitoids with the ovipositor removed, and mortality increased when attacked by untreated parasitoids. On this basis, the authors further studied the responses of caterpillars to volatiles from plants attacked by the larvae of the same species and volatiles from plants on which the caterpillars were themselves attacked by parasitic wasps. Lack of OR mediated olfactory inputs prevents caterpillars from finding suitable food sources and from choosing spaces free of enemies.

Strengths:

The findings help to understand the important role of olfaction in caterpillar feeding and predator avoidance, highlighting the importance of odorant receptor genes in shaping ecological interactions.

Weaknesses:

There are the following major concerns:

(1) Possible non-targeted effects of Orco knockout using CRISPR/Cas9 should be analyzed and evaluated in Materials and Methods and Results

(2) Figure 1E: Only one olfactory receptor neuron was marked in WT. There are at least three olfactory sensilla at the top of the maxillary palp. Therefore, to explain the loss of Orco expressing neurons in the mutant (Figure 1F), a more rigorous explanation of the photo is required.

(3) In Figure 1G, H, the four glomeruli circled by dotted lines: their corresponding relationship between the two figures needs to be further clarified.

(4) Line 130: Since the main topic in this study is the olfactory system of larvae, the experimental results of this part are all about antennal electrophysiological responses, mating frequency and egg production of female and male adults of wild type and Orco KO mutant, it may be considered to include this part in the supplementary files. It is better to include some data about the olfactory responses of larvae.

(5) Line 166: The sentences in the text is about the choice test between " healthy plant vs. infested plant", while in Fig 3C, it is "infested plant vs. no plant". The content in the text does not match the figure.

(6) Lines 174-178: Fig 3A showed that the body weight of Orco KO larvae in the absence of parasitic wasps also decreased compared with that of WT. Therefore, in the experiments of Fig 3A and E, the difference in the body weight of Orco KO larvae in the presence or absence of parasitic wasps without ovipositors should also be compared. The current data cannot determine the reduced weight of KO mutant is due to the Orco knockout or the presence of parasitic wasps.

(7) Lines 179-181: Fig 3F show that the survival rate of larvae of Orco KO mutant decreased in the presence of parasitic wasps, and the difference in survival rate of larvae of WT and Orco KO mutant in the absence of parasitic wasps should also be compared. The current data cannot determine whether the reduced survival of the KO mutant is due to the Orco knockout or the presence of parasitic wasps.

(8) In Figure 4B, why do the compounds tested had no volatiles derived from plants? Cruciferous plants have the well-known mustard bomb. In the behavioral experiments the larvae responses to ITC compounds were not included, which is suggested to be explained in the discussion section.

(9) The custom-made setup and the relevant behavioral experiments in Fig 4C needs to be described in detail (Line 545).

(10) Materials and Methods Line 448: 10 μL paraffin oil should be used for negative control.

Comments on revised version:

The authors have replied my concerns and made revisions accordingly.

# Author comment: Author response

**DOI:** 10.7554/eLife.105585.3.sa2

**Authors:** Qi Wang, Yufei Jia, Hans Smid, Berhane Weldegergis, Liana Greenberg, Maarten Jongsma, Marcel Dicke, Alexander Haverkamp

**Affiliations:** Wageningen University and Research Wageningen Netherlands; Wageningen University and Research Wageningen Netherlands; Wageningen University Wageningen Netherlands; Wageningen University and Research Wageningen Netherlands; Wageningen University and Research Wageningen Netherlands; Wageningen University and Research Wageningen Netherlands; Wageningen University Wageningen Netherlands; Wageningen University and Research Wageningen Netherlands

The following is the authors’ response to the original reviews.

**Public Reviews:**

**Reviewer #1 (Public review):**

Summary:

The manuscript focuses on the olfactory system of Pieris brassicae larvae and the importance of olfactory information in their interactions with the host plant Brassica oleracea and the major parasitic wasp Cotesia glomerata. The authors used CRISPR/Cas9 to knockout odorant receptor coreceptors (Orco), and conducted a comparative study on the behavior and olfactory system of the mutant and wild-type larvae. The study found that Orco-expressing olfactory sensory neurons in antennae and maxillary palps of Orco knockout (KO) larvae disappeared, and the number of glomeruli in the brain decreased, which impairs the olfactory detection and primary processing in the brain. Orco KO caterpillars show weight loss and loss of preference for optimal food plants; KO larvae also lost weight when attacked by parasitoids with the ovipositor removed, and mortality increased when attacked by untreated parasitoids. On this basis, the authors further studied the responses of caterpillars to volatiles from plants attacked by the larvae of the same species and volatiles from plants on which the caterpillars were themselves attacked by parasitic wasps. Lack of OR-mediated olfactory inputs prevents caterpillars from finding suitable food sources and from choosing spaces free of enemies.

Strengths:

The findings help to understand the important role of olfaction in caterpillar feeding and predator avoidance, highlighting the importance of odorant receptor genes in shaping ecological interactions.

Weaknesses:

There are the following major concerns:

(1) Possible non-targeted effects of Orco knockout using CRISPR/Cas9 should be analyzed and evaluated in Materials and Methods and Results.

Thank you for your suggestion. In the Materials and Methods, we mention how we selected the target region and evaluated potential off-target sites by Exonerate and CHOPCHOP. Neither of these methods found potential off-target sites with a more-than-17-nt alignment identity. Therefore, we assumed no off-target effect in our Orco knockout. Furthermore, we did not find any developmental differences between wildtype and knockout caterpillars when these were reared on leaf discs in Petri dishes (Fig S4). We will further highlight this information on the off-target evaluation in the Results section.

(2) Figure 1E: Only one olfactory receptor neuron was marked in WT. There are at least three olfactory sensilla at the top of the maxillary palp. Therefore, to explain the loss of Orcoexpressing neurons in the mutant (Figure 1F), a more rigorous explanation of the photo is required.

Thank you for pointing this out. The figure shows only a qualitative comparison between WT and KO and we did not aim to determine the total number of Orco positive neurons in the maxillary palps or antennae of WT and KO caterpillars, but please see our previous work for the neuron numbers in the caterpillar antennae (Wang et al., 2024). We did indeed find more than one neuron in the maxillary palps, but as these were in very different image planes it was not possible to visualize them together. However, we will add a few sentences in the Results and Discussion section to explain the results of the maxillary palp Orco staining.

(3) In Figure 1G, H, the four glomeruli are circled by dotted lines: their corresponding relationship between the two figures needs to be further clarified.

Thank you for pointing this out. The four glomeruli in Figure 1G and 1H are not strictly corresponding. We circled these glomeruli to highlight them, as they are the best visualized and clearly shown in this view. In this study, we only counted the number of glomeruli in both WT and KO, however, we did not clarify which glomeruli are missing in the KO caterpillar brain. We will further clarify this in the figure legend.

(4) Line 130: Since the main topic in this study is the olfactory system of larvae, the experimental results of this part are all about antennal electrophysiological responses, mating frequency, and egg production of female and male adults of wild type and Orco KO mutant, it may be considered to include this part in the supplementary files. It is better to include some data about the olfactory responses of larvae.

Thank you for your suggestion. We do agree with your suggestion, and we will consider moving this part to the supplementary information. Regarding larval olfactory response, we unfortunately failed to record any spikes using single sensillum recordings due to the difficult nature of the preparation; however we do believe that this would be an interesting avenue for further research.

(5)Line 166: The sentences in the text are about the choice test between " healthy plant vs. infested plant", while in Fig 3C, it is "infested plant vs. no plant". The content in the text does not match the figure.

Thank you for pointing this out. The sentence is “We compared the behaviors of both WT and Orco KO caterpillars in response to clean air, a healthy plant and a caterpillar-infested plant”. We tested these three stimuli in two comparisons: healthy plant vs no plant, infested plant vs no plant. The two comparisons are shown in Figure 3C separately. We will aim to describe this more clearly in the revised version of this manuscript.

(6) Lines 174-178: Figure 3A showed that the body weight of Orco KO larvae in the absence of parasitic wasps also decreased compared with that of WT. Therefore, in the experiments of Figure 3A and E, the difference in the body weight of Orco KO larvae in the presence or absence of parasitic wasps without ovipositors should also be compared. The current data cannot determine the reduced weight of KO mutant is due to the Orco knockout or the presence of parasitic wasps.

Thank you for pointing this out. We did not make a comparison between the data of Figures 3A and 3E since the two experiments were not conducted at the same time due to the limited space in our BioSafety III greenhouse. We do agree that the weight decrease in Figure 3E is partly due to the reduced caterpillar growth shown in Figure 3A. However, we are confident that the additional decrease in caterpillar weight shown in Figure 3E is mainly driven by the presence of disarmed parasitoids. To be specific, the average weight in Figure 3A is 0.4544 g for WT and 0.4230 g for KO, KO weight is 93.1% of WT caterpillars. While in Figure 3E, the average weight is 0.4273 g for WT and 0.3637 g for KO, KO weight is 85.1% of WT caterpillars. We will discuss this interaction between caterpillar growth and the effect of the parasitoid attacks more extensively in the revised version of the manuscript.

(7) Lines 179-181: Figure 3F shows that the survival rate of larvae of Orco KO mutant decreased in the presence of parasitic wasps, and the difference in survival rate of larvae of WT and Orco KO mutant in the absence of parasitic wasps should also be compared. The current data cannot determine whether the reduced survival of the KO mutant is due to the Orco knockout or the presence of parasitic wasps.

We are happy that you highlight this point. When conducting these experiments, we selected groups of caterpillars and carefully placed them on a leaf with minimal disturbance of the caterpillars, which minimized hurting and mortality. We did test the survival of caterpillars in the absence of parasitoid wasps from the experiment presented in Figure 3A, although this was missing from the manuscript. There is no significant difference in the survival rate of caterpillars between the two genotypes in the absence of wasps (average mortality WT = 8.8 %, average mortality KO = 2.9 %; P = 0.088, Wilcoxon test), so the decreased survival rate is most likely due to the attack of the wasps. We will add this information to the revised version of the manuscript.

(8) In Figure 4B, why do the compounds tested have no volatiles derived from plants? Cruciferous plants have the well-known mustard bomb. In the behavioral experiments, the larvae responses to ITC compounds were not included, which is suggested to be explained in the discussion section.

Thank you for the suggestion. We assume you mean Figure 4D/4E instead of Figure 4B. In Figure 4B, many of the identified chemical compounds are essentially plant volatiles, especially those from caterpillar frass and caterpillar spit. In Figure 4D/4E, most of the tested chemicals are derived from plants. But indeed, we did not include ITCs, based on information from the EAG results in Figures 2A & 2B. Butterfly antennae did not respond strongly to ITCs, so we did not include ITCs in the larval behavioural tests. Instead, the tested chemicals in Figure 4D/4E either elicit high EAG responses of butterflies or have been identified as “important” by VIP scores in the chemical analyses. In the EAG results of *Plutella xylostella* (Liu et al., 2020), moths responded well to a few ITCs, the tested ITCs in our study are actually adopted from this study except for those that were not available to us. However, butterflies did not show a strong response to the tested ITCs; therefore, we did not include ITCs because we expected that *Pieris brassicae* caterpillars are not likely to show good responses to ITCs. We will add this explanation to the revised version of our manuscript.

(9) The custom-made setup and the relevant behavioral experiments in Figure 4C need to be described in detail (Line 545).

We will add more detailed descriptions for the setup and method in the Materials and Methods.

(10) Materials and Methods Line 448: 10 μL paraffin oil should be used for negative control.

Thank you for pointing this out. We used both clean filter paper and clean filter paper with 10 μL paraffin oil as negative controls, but we did not find a significant difference between the two controls. Therefore, in the EAG results of Figure 2A/2B, we presented paraffin oil as one of the tested chemicals. We will re-run our statistical tests with paraffin oil as negative control, although we do not expect any major differences to the previous tests.

**Reviewer #2 (Public review):**

Summary:

This manuscript investigated the effect of olfactory cues on caterpillar performance and parasitoid avoidance in Pieris brassicae. The authors knocked out Orco to produce caterpillars with significantly reduced olfactory perception. These caterpillars showed reduced performance and increased susceptibility to a parasitoid wasp.

Strengths:

This is an impressive piece of work and a well-written manuscript. The authors have used multiple techniques to investigate not only the effect of the loss of olfactory cues on host-parasitoid interactions, but also the mechanisms underlying this.

Weaknesses:

(1) I do have one major query regarding this manuscript - I agree that the results of the caterpillar choice tests in a y-maze give weight to the idea that olfactory cues may help them avoid areas with higher numbers of parasitoids. However, the experiments with parasitoids were carried out on a single plant. Given that caterpillars in these experiments were very limited in their potential movement and source of food - how likely is it that avoidance played a role in the results seen from these experiments, as opposed to simply the slower growth of the KO caterpillars extending their period of susceptibility? While the two mechanisms may well both take place in nature - only one suggests a direct role of olfaction in enemy avoidance at this life stage, while the other is an indirect effect, hence the distinction is important.

We do agree with your comment that both mechanisms may be at work in nature and we do address this in the Discussion section. In our study, we did find that wildtype caterpillars were more efficient in locating their food source and did grow faster on full plants than knockout caterpillars. This faster growth will enable wildtype caterpillars to more quickly outgrow the life-stages most vulnerable to the parasitoids (L1 and L2). The olfactory system therefore supports the escape from parasitoids indirectly by enhancing feeding efficiency directly.

Figure 3D shows that WT caterpillars prefer infested plants without parastioids to infested plants with parasitoids. In addition, we observed that caterpillars move frequently between different leaves. Therefore, we speculate that WT caterpillars make use of volatiles from the plant or from (parasitoid-exposed) conspecifics via their spit or faeces to avoid parts of the plant potentially attracting natural enemies. Knockout caterpillars are unable to use these volatile danger cues and therefore do not avoid plant parts that are most attractive to their natural enemies, making KO caterpillars more susceptible and leading to more natural enemy harassment. Through this, olfaction also directly impacts the ability of a caterpillar to find an enemy-free feeding site.

We think that olfaction supports the enemy avoidance of caterpillars via both these mechanisms, although at different time scales. Unfortunately, our analysis was not detailed enough to discern the relative importance of the two mechanisms we found. However, we feel that this would be an interesting avenue for further research. Moreover, we will sharpen our discussion on the potential importance of the two different mechanisms in the revised version of this manuscript.

(2) My other issue was determining sample sizes used from the text was sometimes a bit confusing. (This was much clearer from the figures).

We will revise the sample size in the text to make it more clear.

(3) I also couldn't find the test statistics for any of the statistical methods in the main text, or in the supplementary materials.

Thank you for pointing this out. We will provide more detailed test statistics in the main text and in the supplementary materials of the revised version of the manuscript.

**Recommendations for the authors:**

**Reviewer #1 (Recommendations for the authors):**

(1) Abstract

Line 24: "optimal food plant" should be changed to "optimal food plants"

Thank you for the suggestion, we will revise it.

(2) Introduction

Lines 44-46: The sentence should be rephrased.

Thank you for the suggestion, we will revise it.

Line 50: "are" should be changed to "is".

Thank you for the suggestion, we will revise it.

Lines 57 and 58: Please provide the Latin names of "brown planthoppers" and "striped stem borer".

Thank you for the suggestion, we will revise it.

Line 85: "investigate the influence of odor-guided behavior by this primary herbivore on the next trophic levels"; similarly, Line 160: "investigate if caterpillars could locate the optimal host-plant when supplied with differently treated plants". These sentences are not very accurate in describing the relevant experiments. A: Thank you for the suggestion, we will revise them.

**Reviewer #2 (Recommendations for the authors):**

(1) L53 Remove the "the" from "Under the strong selection pressure"

Thank you for the suggestion, we will revise it.

(2) L80 I suggest adding a reference for the spitting behaviour, e.g. Muller et al 2003.

Thank you for the suggestion, we will add it.

(3) L89 establishing a homozygous KO insect colony.

Thank you for the suggestion, we will revise it.

(4) L107 perhaps this goes against the journal style but I always like to see acronyms explained the first time they are used.

Thank you for the suggestion, we will try to make it more understandable.

(5) L146-148 sentence difficult to read - consider rephrasing.

Thank you for the suggestion, we will revise it.

(6) L230 do you mean still produce? Rather than still reproduce?

Thank you for the suggestion, we will revise it.

(7) L233 missing an and before "a greater vulnerability to the parasitoid wasp".

Thank you for pointing this out, we will revise it.

(8) L238 malfunctional is a strange word choice.

Thank you for pointing this out, we will revise it.

(9) L181 - can the authors confirm that this lower survival was due to parasitism by the wasps?

This question is similar to Q(7) of Reviewer 1, so we quote our answer for Q(7) here:

When conducting these experiments, we selected groups of caterpillars and carefully placed them on a leaf with minimal disturbance of the caterpillars, which minimized hurting and mortality. We did test the survival of caterpillars in the absence of parasitoid wasps from the experiment presented in Figure 3A, although this was missing from the manuscript. There is no significant difference in the survival rate of caterpillars between the two genotypes in the absence of wasp (average mortality WT = 8.8 %, average mortality KO = 2.9 %; P = 0.088, Wilcoxon test), so the decreased survival rate is most likely due to the attack of the wasps. We will add this information to the revised version of the manuscript.

(10) L474 - has it been tested if wasps still behave similarly after their ovipositor has been removed?

Thank you for pointing out this issue. We did not strictly compare if disarmed and untreated wasps have similar behaviors. However, we did observe if disarmed wasps can actively move or fly after recovering from anesthesia before releasing into a cage, otherwise we would replace with another active one.
